# Indications and opportunities for transsplenic access to treat portal venous and portosystemic pathology: A report of 2 cases and review of the literature

**DOI:** 10.1016/j.radcr.2025.12.053

**Published:** 2026-01-24

**Authors:** Trinity Puno, Joshua Levy, Daniel Villegas, Marvi Moreno, Ryan Rimer, Brandon Chen

**Affiliations:** a Rocky Vista University - Montana College of Osteopathic Medicine, 4130 Rocky Vista Way, Billings, MT 59106, USA; b Transitional Year Program, Southern Hills Hospital & Medical Center, 9300 W Sunset Rd, Las Vegas, NV 89148, USA; c Department of General Surgery, University Medical Center of Southern Nevada, 1800 W Charleston Blvd, Las Vegas, NV 89102, USA; d Division of Vascular & Interventional Radiology, University of Texas Southwestern Medical Center, 5323 Harry Hines Blvd, Dallas, TX 75390, USA; e Department of Vascular & Interventional Radiology, University Medical Center of Southern Nevada, 1800 W Charleston Blvd, Las Vegas, NV 89102, USA

**Keywords:** Transsplenic access, Portal vein, Splenic vein, Angioplasty, Varices, TIPS

## Abstract

Transsplenic access has emerged as a valuable technique for portal venous interventions when conventional transjugular or transhepatic approaches are challenging due to prohibitive anatomy. We briefly summarize the literature on how transsplenic access came to be and present 2 unique cases that highlight the importance of transsplenic access in facilitating transjugular intrahepatic portosystemic shunt creation in complex portal venous disease. The first case describes a 68-year-old male cirrhotic patient with extensive acute-on-chronic portal vein thromboses in whom transsplenic access enabled portal vein thrombectomy and successful portosystemic shunt placement. The second case involves a 60-year-old female patient with chronic portal vein thrombosis and cavernous transformation, where a combined transsplenic–transhepatic “body floss” technique allowed recanalization and subsequent portosystemic shunt creation.

## Introduction

Transsplenic access (TSA) for portal vein interventions has evolved significantly over the past few decades, providing an alternative route for managing various portal vein pathologies when traditional approaches are technically challenging or unsuccessful.

The initial use of TSA was described in 1951 primarily for diagnostic purposes, such as splenoportography, to visualize the portal venous system [1,2]. TSA was performed using individualized techniques until a more standardized approach was described by Kreel in 1970 to increase the safety of the procedure and consistency of the results [3]. Initially, the incidence of bleeding secondary to splenic lacerations and trauma was a major concern, resulting in the abandonment of this technique as the rise of abdominal computed tomography (CT) came to be widely adopted [4,5]. In 1978, TSA was reintroduced as a safe procedure with less bleeding complications when the access site was occluded using Gelfoam (Pfizer, New York, NY, USA) [6]. This has since been expanded upon to embolize the access site with other embolic agents including liquid embolics, detachable coils, vascular plugs, or a combination of the aforementioned materials under fluoroscopic and/or ultrasound guidance [1].

Since the early 2000′s, TSA has expanded as a therapeutic technique to treat multiple portal vein (PV) pathologies including PV recanalization from thromboembolic occlusion, portosystemic variceal embolization, and even portosystemic venous shunt embolization due to difficult or variant anatomy. In 2013, a study by Zhu et al. reported a 96% technical success rate in portal vein catheterization via TSA with major bleeding observed in only 6.5% of patients [7]. A total of 44 patients who required gastroesophageal variceal embolization were successfully treated via TSA. Two failures were due to inaccessible intrasplenic veins likely related to small size [7].

A study in 2015 by Habib et al. [8] showed that TSA for portal vein recanalization-transjugular intrahepatic portosystemic shunt (PVR-TIPS) was successful in 11 patients without major complications and improved transplant candidacy in patients with liver cirrhosis and end stage liver disease. These patients were previously denied transplant listing due to the presence of a main portal vein thrombosis, but postprocedurally, 3 patients underwent successful liver transplantation. Minor adverse events included fever or encephalopathy and occurred in only 2 patients [8]. Transsplenic PVR-TIPS was additionally performed using a balloon puncture technique in a study by Meine et al. [9] that was technically successful in 12 out of 14 patients (86%), with only 2 patients unable to receive stent graft placement due to undetectable intrahepatic PV branches. This technique used a balloon catheter that is inflated inside the occluded portion of the PV which would be used as a fluoroscopic target for the TIPS needle. Complications including subcapsular splenic hematoma, spontaneous bacterial peritonitis, and access site bleeding occurred in only 4 patients [9].

As of 2022, more novel TSA techniques have been described for various unique indications including direct retropancreatic splenic vein puncture after splenectomy to treat PV thrombosis, jejunal varix embolization after pancreaticoduodenectomy, and gastric varix embolization in the setting of malignant left-sided portal hypertension [[10], [11], [12]]. TSA has also been used in portosystemic venous shunt embolization. A novel study by Villegas et al. [13] in 2024 demonstrated 7 different intrahepatic portosystemic venous shunts that were successfully embolized via the TSA approach in a patient who had no evidence of liver disease or trauma. The transhepatic and transjugular approaches were not pursued in this case due to the pronounced angulation and tortuosity of each shunt, which rendered navigation via this technique exceedingly challenging.

While transjugular and transhepatic routes are more commonly used, proficiency in the transsplenic approach broadens access options for select patient populations and complex variant anatomy. However, because clinical reports on this technique remain scarce, there are currently no standardized guidelines defining its indications or procedural best practices. Herein, we present 2 cases demonstrating successful use of the transsplenic approach for TIPS creation in hopes of broadening the scope of transsplenic access for treatment of portosystemic disease.

### Case presentation

**Fig. 1:**
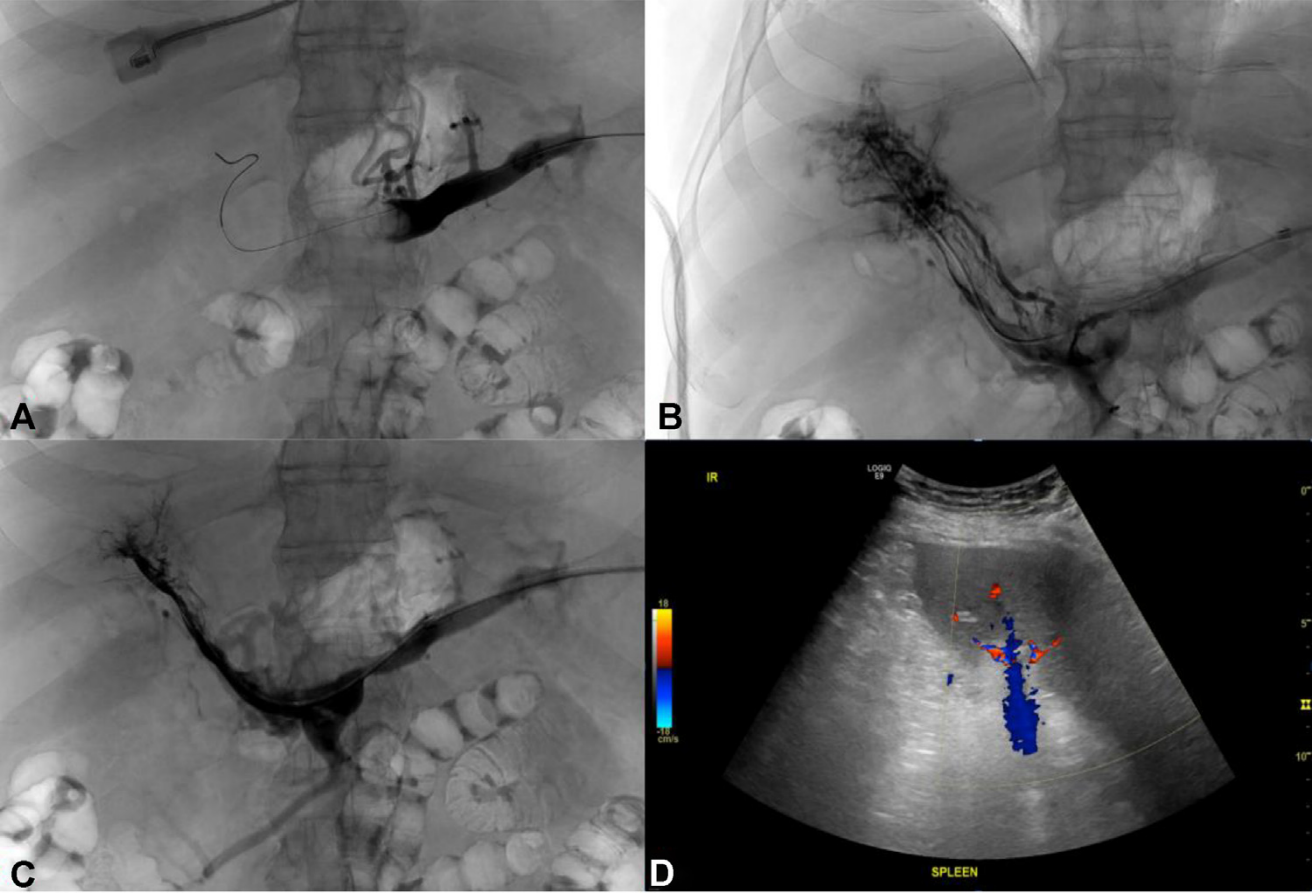
Portal vein thrombus recanalization using TSA. (A) The splenic vein was accessed using a 21-gauge micropuncture needle followed by a 0.018 inch guidewire and eventual transitional dilator once the needle was removed. Next, an 8 French sheath was selected and placed into the splenic vein, and venography of the splenic and portal veins was performed, demonstrating large, nonocclusive portal vein and splenic vein thrombi. (B) After identifying thrombi within the portal and splenic veins, an 8 French Aspirex mechanical thrombectomy device (Bard Peripheral Vascular, Tempe, Arizona, USA) was advanced over a 0.035 inch guidewire to perform mechanical aspiration thrombectomy. Repeat venography demonstrated improved vascular flow. (C) Repeat thrombectomy was done to further decrease clot burden and improve portal and splenic vein flow, allowing for a TIPS stent-graft to be placed and (D) Scout ultrasound images of the spleen showing patent splenic vein branches within the splenic hilum.

**Fig. 2:**
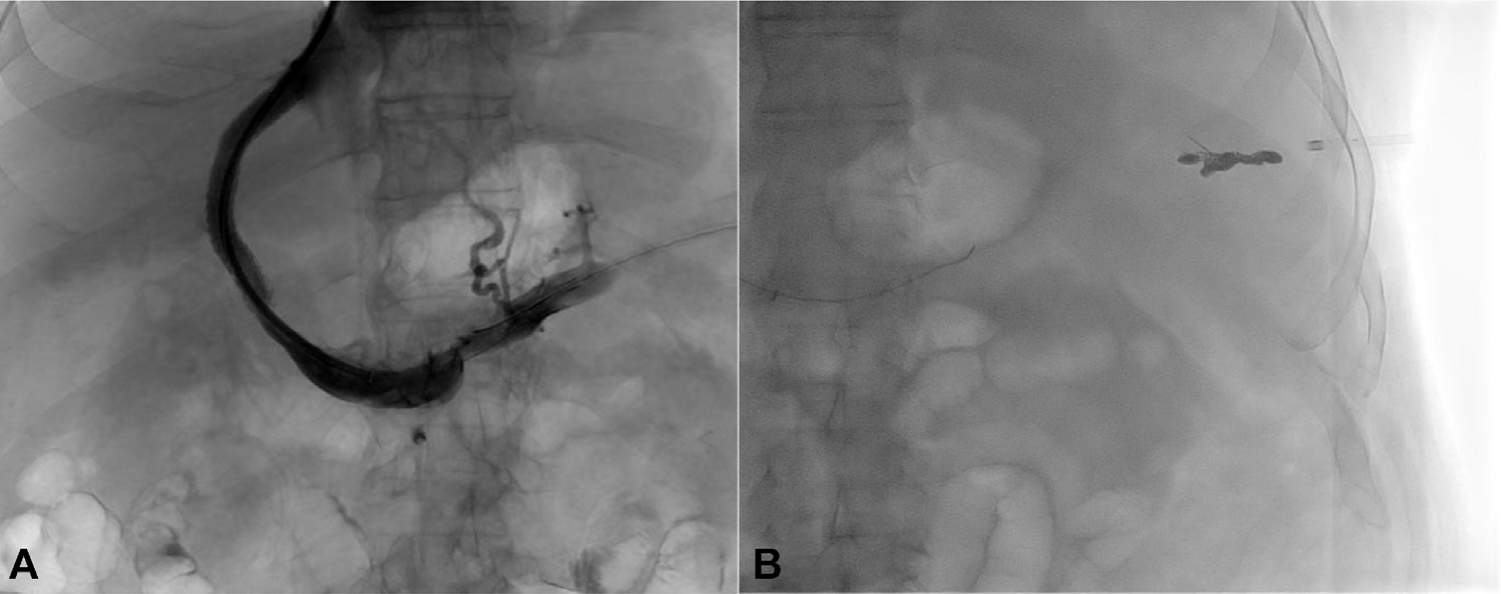
TIPS placement in a cirrhotic patient with esophageal varices. (A) Using fluoroscopic guidance, a multipurpose angiographic catheter was advanced into the right hepatic vein. A snare was placed into the right portal vein via TSA. The catheter was exchanged for a Colapinto needle (Cook, Bloomington, IN, USA). Passes were made across the liver parenchyma until the right portal vein was entered. A 0.035 inch guidewire followed by the multipurpose catheter was advanced into the portal vein which was confirmed by contrast injection. A marking pigtail catheter was then advanced into the portal vein. Simultaneous portal and hepatic venography was done to measure the length of the transhepatic tract. The catheter was exchanged over an extra-stiff guidewire for an 8 mm balloon which was used to predilate the parenchymal tract. An 8 cm covered by 10 mm, 2 cm uncovered Viatorr stent-graft (W.L. Gore & Associates, Phoenix, AZ, USA) was advanced and deployed across the shunt in routine fashion without complication. The stent graft was dilated using a 10 mm balloon. (B) The transsplenic access tract was successfully embolized postprocedurally using a series of detachable coils. After tract embolization, the 8 French sheath was removed from the spleen. The catheters were removed, and hemostasis was achieved at the access site by manual compression.

Case 1**:** A 68 year-old male with a history of hepatitis C, hyperlipidemia, esophageal varices, liver cirrhosis, and prior deep vein thromboses presented for hematemesis. Over the course of 6 months, he reported intermittently worsening abdominal pain and distention after receiving a new diagnosis of cirrhosis. Prior to arrival, he had 1 large episode of hematemesis and 1 black and tarry stool. Abdominal ultrasound with doppler demonstrated a large left portal vein thrombus, and abdominal CT imaging with intravenous (IV) contrast demonstrated occlusive thrombi in the right, left, and main portal veins extending into the portal confluence, superior aspect of the superior mesenteric vein, and central portion of the splenic vein. Bilateral venous doppler ultrasounds also showed chronic thromboses in the left superficial femoral, popliteal, and peroneal veins for which an inferior vena cava (IVC) filter was placed prior to TSA for thrombectomy. Fig. 1, Fig. 2 highlight the intraoperative ultrasound for TSA as well as thrombectomy and eventual TIPS stent graft placement.

**Fig. 3:**
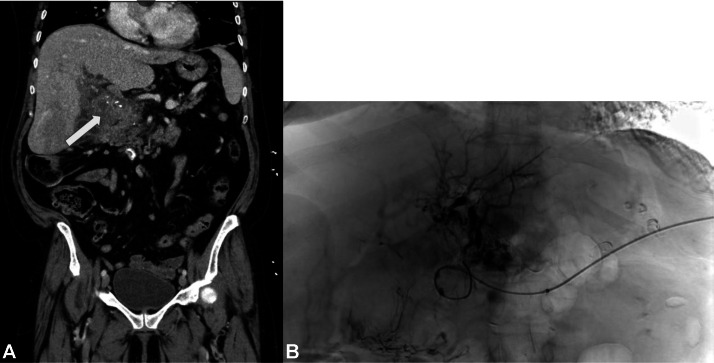
Portal cavernoma with collateralization. (A) Coronal CT portal venous phase image demonstrating PV thrombosis (white arrow) with numerous vascular collaterals, consistent with a portal cavernoma. A thin rim of contrast persists around the central filling defect, known as the polo mint sign. Medial to the arrow, calcifications of the porta hepatis can be visualized and (B) Splenic venogram showing extensive cavernous transformation of the PV with collateralization. Vertebral curve catheter and wires used could not successfully traverse the chronic PV thrombosis despite multiple attempts.

**Fig. 4:**
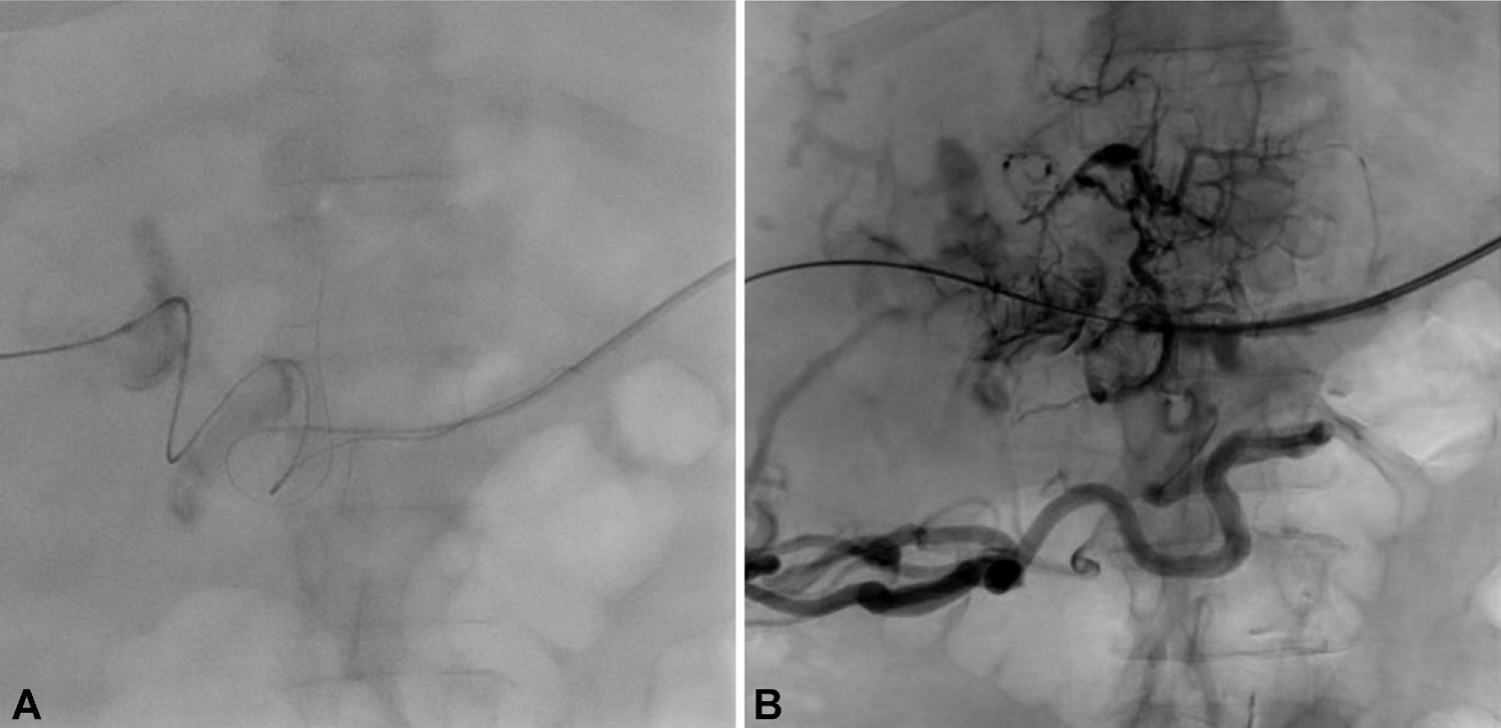
Recanalization of cavernous PV with TSA approach. (A) Using the transsplenic approach catheter as a guide, the thrombosed cavernous PV was recanalized using a transhepatic approach, and a 0.035 inch guidewire was successfully advanced into the splenic vein and (B) The wire was then snared and externalized through the splenic vein access (body floss), significantly reducing the tortuosity of the cavernous portal venous system.

**Fig. 5:**
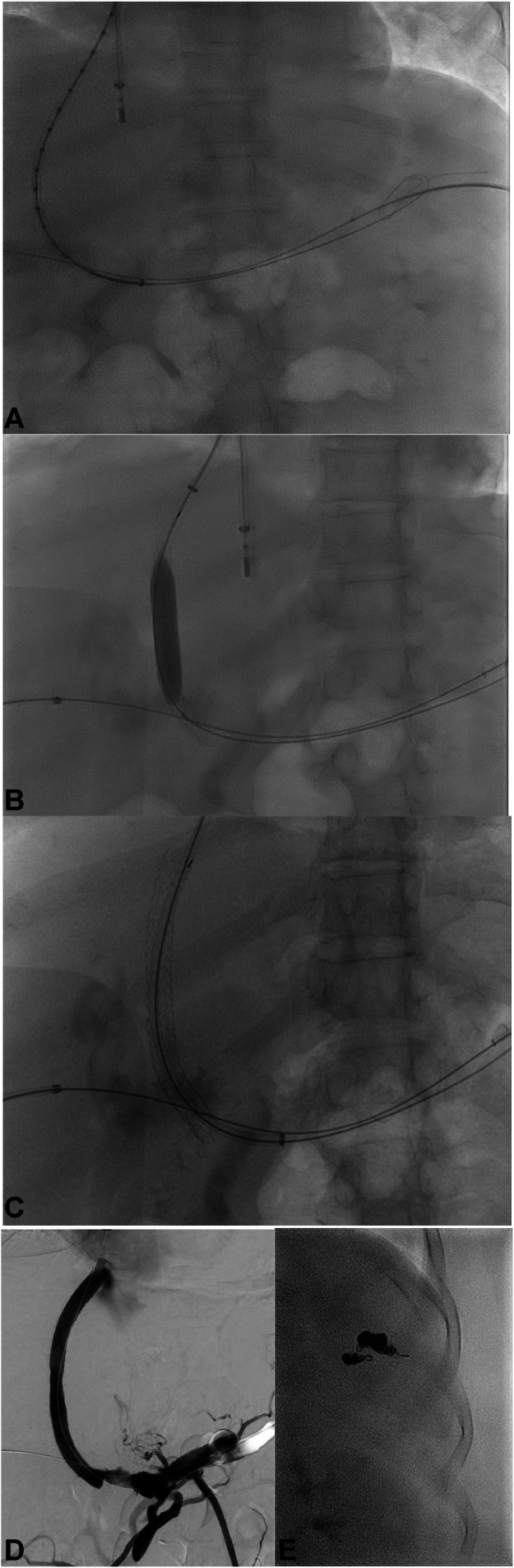
TIPS insertion in a patient with portal biliopathy facilitated by TSA. (A) Following tract dilation, a vascular sheath was advanced into the PV and a portal venogram was performed with a marking pigtail catheter. (B) A 10 mm by 8 cm covered, 2 cm uncovered Viatorr stent was then deployed across the shunt in standard fashion. Angioplasty of the stent was performed with a 10 mm balloon. (C) The portal venous end of the TIPS was extended with a 10 mm x 39 mm Wallstent stent (Boston Scientific, Marlborough, MA, USA). The hepatic venous end of the TIPS was extended with a 10 mm x 50 mm Viabahn stent (W.L. Gore & Associates, Flagstaff, AZ, USA). (D) Repeat portal venogram demonstrated inline flow from splenic vein into TIPS and (E) At the end of the procedure, all wires, catheters, and vascular sheaths were removed. The splenic access tract was embolized using multiple endovascular coils to prevent splenic hemorrhage postoperatively.

Case 2: A 60 year-old female with PV cavernous transformation and portal biliopathy presented with chronic abdominal pain status post multiple endoscopic interventions with gastroenterology. Abdominal CT with IV contrast shown in Fig. 3 demonstrated cavernous transformation of the portal vein with portal vein thrombosis in the main portal, left, and right portal veins. After failed attempts to recanalize the portal vein via TSA alone, Fig. 3, Fig. 4, Fig. 5 demonstrate a combined transsplenic-transhepatic approach that was used to establish through-and-through access for thrombectomy. Using the “body floss” technique, the thrombosed portal vein was successfully traversed and dilated. TIPS creation was achieved using a Viatorr stent with extensions, followed by mechanical thrombectomy and balloon maceration.

For case 1, postprocedural imaging demonstrated decreased clot burden with reduction in filling esophageal varices after embolization. The PV thrombus was consistent with metastatic hepatocellular carcinoma. The patient was subsequently discharged to an acute rehabilitation facility and was later lost to follow-up with the interventional radiology service. For case 2, the patient was managed post-procedurally with anticoagulation and medical therapy. The patient remained in the medical intensive care unit for management of the PV thrombus and subsequently received a tracheostomy and percutaneous endoscopic gastrostomy placement. They were eventually transferred to a skilled nursing facility after being medically stabilized.

## Discussion

We believe our 2 cases demonstrate the feasibility and safety of TSA for successful TIPS creation in complex portal venous disease. Transjugular and transhepatic approaches remain the standard access approaches for TIPS procedures, but TSA has emerged over the past few decades as a valuable alternative when traditional access routes prove to be technically challenging [14].

Current evidence indicates that TSA achieves quite high technical success rates that range from 86% to 96.7% for portal venous interventions. A recent single-center study of 30 unique patients undergoing TSA for portal intervention showed a 96.7% technical success rate for catheterization with no significant bleeding events from the splenic parenchyma, splenic vein, or percutaneous access point when adequate tract closure techniques were used [1,9]. Taken together, these findings demonstrate that TSA offers high technical success rates with a favorable safety profile as complications were limited to minor, spontaneously resolving issues of flank pain from small subcapsular hematomas or mild pleural effusions [1].

The American Association for the Study of Liver Diseases (AASLD) 2024 practice guidance update stated that acute or chronic bland PV thrombosis is not an absolute contraindication to TIPS [15]. However, an important caveat is that specialized technical skills may be required, including TSA. While the feasibility of TIPS placement diminishes when patients have a concomitant portal cavernoma or chronic PV thrombosis, novel TSA access routes are able to achieve TIPS placement and recanalization in 90%-100% of patients, which allows for a wider access for TIPS and even liver transplantation if indicated at a later stage [16].

Our cases necessitated TIPS via the TSA approach due to extensive, tortuous PV thromboses, and both cases ultimately resulted in successful TIPS creation. Additional primary indications for choosing TSA over traditional access routes include situations in which the transhepatic window is inaccessible due to occlusion, inability to detect PV branches, or in post-liver transplant patients where preserving the liver graft is critical [14]. TSA has also shown to be valuable in patients undergoing liver resection when the anticipated liver remnant may be insufficient to function normally. In such cases, PV embolization is performed to promote growth of the future liver remnant. Most commonly, the transhepatic route is accessed for PV embolization. This approach requires navigating narrow and acute angles with reverse curve catheters. Interestingly, Sarwar et al. [17] were able to demonstrate that the transsplenic route permits straightforward entry into the ipsilateral PV branches while simultaneously decreasing the risk of injury to the remaining liver tissue. Thus, TSA is a multifaceted technique that could prove useful as another technique for interventional radiologists to employ when working with trauma/transplant patients.

The existing literature on TSA is growing but still largely composed of small series and case reports. One retrospective series reported a 98.4% (60/61) success rate in portal vein recanalization using TSA with fewer adverse events when compared to the transhepatic approach [18]. Comparative data between TSA and alternative strategies (eg, transmesenteric or surgical bypass) are limited. Ongoing and future studies may define optimal selection criteria (eg, when to choose TSA first), best embolic agents for tract closure, and long-term outcomes (eg, portal patency, rebleeding rates). Multicenter studies could help clarify complication rates in broader everyday practice.

## Conclusion

The cases presented here contribute to the growing body of literature supporting TSA as a valuable approach when standard access routes may pose a challenge or are technically unfeasible. Safety data that is currently available has acceptable complication rates comparable to transhepatic access and minimal risk for major bleeding episodes with meticulous tract embolization. TSA offers a direct and anatomically advantageous route to the portal venous system, allowing for a wide range of diagnostic and therapeutic interventions. As familiarity grows and operator experience continues to steadily increase, TSA may allow more patients to benefit from minimally invasive portal therapies that would otherwise be unreachable.

## Data availability statement

Data may be obtained from the corresponding author.

## Patient consent

In accordance with the Institutional Review Board policy of the hospital at which the patients were treated, written informed consent for publication was obtained for the patient described in Case 2. Informed consent could not be obtained for the patient described in Case 1 because the patient was deceased at the time of manuscript preparation with no available next of kin. All data were fully de-identified to protect both patients’ confidentiality.

## Appendix Supplementary materials

Image, application 1
