# Supplementary material for: Indications and opportunities for transsplenic access to treat portal venous and portosystemic pathology: A report of 2 cases and review of the literature
Source: Radiol Case Rep. 2026 Jan 24;21(4):1564–9. doi: 10.1016/j.radcr.2025.12.053 (PMC12860249; doi:10.1016/j.radcr.2025.12.053)
Supplement: Supplementary file 1 [file mmc1.pdf]

Single Case Reports and Case Series
UMC IRB Guidance

Background:
The University Medical Center of Southern Nevada (UMC) Institutional Review Board (IRB) frequently receives queries regarding whether publishing a case report or case series constitutes human subjects research and therefore requires IRB review. Also, many journals now require a letter, or other acknowledgement, from an IRB prior to publication of a case report. Specifically, the journals wish to know whether prospective IRB approval was obtained or was not required for the case or cases described. The UMC IRB has adopted a policy designed to provide guidance on when publications/presentations of case reports constitute research and requires prospective IRB review and approval.

Definitions:
Human Subject(s): As defined by 45CFR46.102(f), means a living individual about whom an investigator (whether professional or student) conducting research obtains

1. Data through intervention or interaction with the individual, or
2. Identifiable private information.

Generalizable Knowledge: Knowledge from which conclusions will be drawn that can be applied to populations outside of the specific study population. This usually includes one or more of the following concepts:

- Knowledge that contributes to a theoretical framework of an established body of knowledge;
- The primary beneficiaries of the research are other researchers, scholars, and practitioners in the field of study;
- Dissemination of the results is intended to inform the field of study (though this alone does not make an activity constitute research "designed to contribute to generalizable knowledge");
- The results are expected to be generalized to a larger population beyond the site of data collection;
- The results are intended to be replicated in other settings.

Research: As defined by 45CFR46.102(d), means a "*systematic investigation*", including research development, testing and evaluation, designed to develop or contribute to "*generalizable knowledge*". Activities which meet this definition constitute research for purposes of this policy, whether or not they are conducted or supported under a program which is considered research for other purposes. For example, some demonstration and service programs may include research activities.

Systematic Investigation: An activity that involves a prospective Research plan which incorporates data collection, either quantitative or qualitative, and data analysis to answer a Research question.

Application:
For UMC IRB purposes, a single case report, case study, and/or case series is a retrospective analysis of one, two, or three clinical cases and generally does not meet the definition of "*research*". Therefore, a single case report **does not** have to be reviewed by the IRB.

A single case report should not be confused with a *case-control study*, which is a type of observational study that meets the definition of human subject research and requires prospective IRB review and approval.

If more than three cases are involved in the analysis, the activity will be considered "*research*" and requires the submission of a new protocol application for prospective IRB review. The review of medical records for publication of "case reports" of typically three or fewer patients is NOT considered "*human subjects*" research and does NOT typically require IRB review and approval because case reporting on a small series of patients does not involve the formulation of a research hypothesis that is subsequently investigated prospectively and systematically for publication or presentation. Reporting or publication is not typically envisioned when one interacts clinically with the subject.

When larger series of patients are being reported, investigators/authors usually begin to ask specific research questions and formal systematic collection of data occurs, moving these activities closer to prospectively designed research.

Researchers are advised to consult with the IRB when uncertainty exists about whether the activity meets the definition of human subjects research.

FINAL

It should also be noted that teaching, and soliciting colleagues' advice on clinical care of a specific patient or groups of patients during presentation of a case at departmental conferences DOES NOT require IRB review. Generalized commentary by a clinician on the outcome of their clinical care of patients in accepted venues for discussion of clinical management is also not considered research requiring IRB review, if there is no prospective research plan, and no formal, systematic and prospective collection of information. This type of communication may occur at hospital or practice meetings, in continuing education venues, or in editorials, where the comments are explicitly identified as personal experience and not formal clinical research.

Many journals now require a letter, or other acknowledgement, from an IRB prior to publication of a case report. If an investigator/author wishes to have the project assessed by the UMC IRB to see if it meets the definition of a single case report, the investigator/author should communicate in writing, the suggested single case report by completing the **Case Report – Investigator/Author Worksheet.** If the project qualifies as a single case report, the UMC IRB will send the investigator/author an acknowledgement letter.

HIPAA Compliance for Case Reports:
Under HIPAA, a case report is an activity to develop information to be shared for medical/educational purposes either internally or externally. Although the use of protected health information to prepare the case report does not require IRB review and approval, the investigator/author of a case report must comply with HIPAA. A quick reference table for HIPAA compliance is included here:

Case Reports: HIPAA Compliance Quick Reference Table

**Use and Disclosure of Patient Information**	**HIPAA Compliance Requirements**
Internal Medical / Educational Purposes (ex: classroom presentation within school)	• Presenter was part of the patient's care team. Mining UMC data for potentially useable past case information is not permitted without prior approval from UMC.<br>• Only minimally necessary information should be used.<br>• De-identified information should be used.<br>• Information cannot be shared outside of the school or hospital.
External Medical / Educational Purposes (ex: case report presentation at a conference or a journal publication)	• Presenter was part of the patient's care team. Mining UMC data for potentially useable past case information is not permitted without prior approval from UMC.<br>• Presenter acquired a HIPAA-compliant patient authorization for the specific use and disclosure of PHI.<br>• If documented patient authorization is not possible, the information must be de-identified in a HIPAA-compliant manner.<br>• Only minimally necessary information should be used.
Publication of Research for Generalizable Knowledge (more than 3 cases)	• Refer to and follow IRB procedures for the protection of human subjects.

Where case report information is to be presented within the school or hospital, HIPAA compliant de-identification is required. Where the case report information is to be shared outside of the school or hospital, the author of the case report should obtain the signed authorization of the subject, or the subject's legally authorized representative, in order to use and present the subject's information in the case report. The authorization should specify which patient identifiers (if any), what condition and treatment information, and the

FINAL

intended uses and disclosures of the information If it is not possible to obtain authorization, then HIPAA-compliant de-identification is required. If the patient information cannot be de-identified, then an authorization is required.

In all cases where the case report information is to be shared outside of the school or hospital, the investigator/author of the case report will obtain the signed authorization of the subject, or the subject's legally authorized representative, in order to use the subject's information in the case report. The authorization should specify which subject identifiers, what condition and treatment information, and the intended uses and disclosures of the information. If it is not possible to obtain authorization, then HIPAA-compliant de-identification is required in accordance with 45 CFR 164.514. In general, in order for information to be considered de-identified, these criteria must be met:

- Health information that does not identify an individual and with respect to which there is no reasonable basis to believe that the information can be used to identify an individual.
- The covered entity, including physician or student, does not have actual knowledge that the information could be used alone or in combination with other information to identify an individual who is a subject of the information.
- The following identifiers of the individual or of relatives, employers, or household members of the individual are removed:
    - Names
    - All geographic subdivisions smaller than a State
    - All elements of dates (except year) for dates directly related to an individual (birth date, admission date, date of death, all ages over 89, etc.)
    - Telephone numbers
    - Fax numbers
    - Email addresses
    - Social security numbers
    - Medical record numbers
    - Health plan beneficiary numbers
    - Account numbers
    - Certificate/license numbers
    - Vehicle identifiers and serial numbers, including license plate numbers
    - Device identifiers and serial numbers
    - Web Universal Resource Locators (URLs)
    - Internet Protocol (IP) addresses
    - Biometric identifiers, including finger and voice prints
    - Full-face photographs and any comparable images
    - Any other unique identifying number, characteristic, or code

If the author strips off all HIPAA identifiers, but the information associated with the subject of the article includes a "unique characteristic" (e.g. photo, illustration, tattoo) which would make it identifiable to the subject, or the author has actual knowledge that the information about the subject could be used alone or in combination with other information to identify the subject, the author must contact the Chief Privacy Officer (Keith Slade at (702) 383-3854) to discuss the required steps to take prior to publication.

FINAL
